# Artificial Intelligence in the Assessment and Grading of Acne Vulgaris: A Systematic Review

**DOI:** 10.3390/jpm15060238

**Published:** 2025-06-06

**Authors:** Daniele Omar Traini, Gerardo Palmisano, Cristina Guerriero, Ketty Peris

**Affiliations:** 1 Dermatologia, Dipartimento Universitario di Medicina e Chirurgia Traslazionale, Università Cattolica del Sacro Cuore, 00168 Rome, Italy; danieleomar.traini01@icatt.it (D.O.T.); cristina.guerriero@policlinicogemelli.it (C.G.); ketty.peris@unicatt.it (K.P.); 2 Dermatologia, Dipartimento Scienze Mediche e Chirurgiche, Fondazione Policlinico Universitario A. Gemelli IRCCS, 00168 Rome, Italy

**Keywords:** acne, artificial intelligence, acne vulgaris, dermatology, computer vision, machine learning

## Abstract

Acne vulgaris is a common dermatological condition, particularly affecting adolescents during critical developmental stages, which may have lasting psychosocial impacts. Traditional assessments, including global severity grading and lesion counting, are limited by subjectivity and time constraints. **Background/Objectives**: This review aims to systematically assess the recent advancements in artificial intelligence (AI) applications for acne diagnosis, lesion segmentation/counting, and severity grading, highlighting the potential of AI-driven methods to improve objectivity, reproducibility, and clinical efficiency. **Methods**: A comprehensive literature search was conducted across PubMed, Scopus, arXiv, Embase, and Web of Science for studies published between 1 January 2017 and 1 March 2025. The search strategy incorporated terms related to “acne” and various AI methodologies (e.g., “neural network”, “deep learning”, “convolutional neural network”). Two independent reviewers screened 345 articles, with 29 studies ultimately meeting inclusion criteria. Data were extracted on study design, dataset characteristics (including internal and publicly available resources such as ACNE04 and AcneSCU), AI architectures (predominantly CNN-based models), and performance metrics. **Results**: While AI-driven models demonstrated promising accuracy, as high as 97.6% in controlled settings, the limited availability of large public datasets, the predominance of data from specific ethnic groups, and the lack of extensive external validation underscore critical barriers to clinical implementation. **Conclusions**: The findings indicate that although AI has the potential to standardize acne assessments, reduce observer variability, and enable self-monitoring via mobile platforms, significant challenges remain in achieving robust, real-world applicability. Future research should prioritize the development of large, diverse, and publicly accessible datasets and undertake prospective clinical validations to ensure equitable and effective dermatological care.

## 1. Introduction

Acne vulgaris is one of the most common dermatological diseases, affecting a significant proportion of the young population, particularly adolescents [1]. Its onset during adolescence, a critical period of individual’s social, emotional, and physical maturation, may negatively impact body image satisfaction, social activities and self-esteem, and often extends into adulthood resulting in impaired quality of life [2].

Accurate measurement and grading of acne are critical for effective management and reproducibility of research [2], but it can be challenging due to spontaneous acne fluctuations and the uneven distribution of lesions.

Traditional acne-severity assessment tools can be categorized into two main approaches: global acne severity grading and lesion counting, both of which have the limitations of subjective assessments and are time-consuming [3]. Global severity grading provides a subjective assessment that compares the clinical presentation with a standard photograph or text description. Currently, the most used acne grading scales include the Investigator Global Assessment (IGA), which features five ordinal grades ranging from 0 to 4 (0, clear; 1, almost clear; 2, mild; 3, moderate; 4, severe) [3] and the Global Acne Severity Scale (GEA), which offers a similar grading scale from 0 to 5 (0, no lesions; 1, nearly no lesions; 2, mild; 3, moderate; 4, severe; 5, very severe) [3].

Another possible approach is lesion counting, exemplified by the Hayashi scale, that evaluates acne severity by counting inflammatory lesions (papules and pustules) on one side of the face and classifies it into four categories: mild (0–5 lesions), moderate (6–20), severe (21–50), and very severe (>50) [3]. Global severity grading tends to be simpler and quicker than lesion counting, but is more operator dependent and less sensitive to disease fluctuation over time. Conversely, lesion counting provides a more objective and reproducible method, but it is often impractical, time-consuming, and does not consider parameters as the pattern distribution, lesions’ size, or the presence of erythema [3]. Both lesion counting and acne severity grading criteria can be further classified into those that consider only the number of lesions and those that incorporate both lesion count and type (e.g., open and closed comedones, papules, pustules, and nodules) [3].

In recent years, the rapid advance of artificial intelligence (AI) and computer vision technologies has opened new avenues for automated and objective acne diagnosis and assessment, through both lesion segmentation/lesion counting and general grading. AI-based systems, trained on vast datasets and sophisticated algorithms to identify and classify acne lesions with high precision, offer the potential for standardized and reproducible ways to assess acne and its response to treatment [4]. The application of AI-driven automated acne-grading algorithms may allow a faster, more precise, reproducible and standardized approach than traditional acne scoring methods, reducing inter- and intra-observer variability [5]. AI algorithms might also lead to self-monitor through mobile applications, assess response to treatments, process enormous amounts of epidemiological data [6], and simplify comparisons across research studies in meta-analyses [7].

This systematic review aims to report recent advancements and implementation of AI applications in acne diagnosis, lesion counting, and severity grading.

## 2. Materials and Methods

We evaluated methodologies such as convolutional neural networks (CNNs), deep residual networks, and hybrid models, focusing on their architectural designs, dataset composition, and performance metrics (e.g., accuracy, sensitivity, specificity). This systematic review was conducted in accordance with the Preferred Reporting Items for Systematic Reviews and Meta-Analyses (PRISMA) 2020 guidelines. A comprehensive search of PubMed, Scopus, arXiv, Embase, and Web of Science was conducted using the following terms: (“acne”) AND (“artificial intelligence” OR “neural network” OR “deep learning” OR “convolutional neural network” OR “transfer learning” OR “machine learning” OR “computer-aided diagnostic” OR “CAD” OR “image classification” OR “image processing” OR “Internet of Things” OR “data mining”) NOT (“Meta-Analysis” OR “Systematic Review”). Studies published between 1 January 2017 and 1 March 2025 were included. Two independent reviewers (D.O.T. and G.P.) screened titles and abstracts to determine the eligibility of studies. We included studies of any design if they enrolled human patients of any age diagnosed with acne vulgaris and evaluated artificial-intelligence or computer-vision algorithms for lesion detection or severity grading. Eligible studies had to benchmark their models against a clinical reference either dermatologist assessments or expert-annotated “ground truth” and report at least one quantitative performance metric (for example, accuracy, area under the receiver-operating-characteristic curve [AUC], Dice coefficient, sensitivity, or specificity). Only retrospective observational investigations published in peer-reviewed journals in English were considered. We excluded conference abstracts, non-English articles, narrative reviews and meta-analyses, and any studies that reused datasets already captured elsewhere to avoid duplication. Because this is a rapidly evolving field, we also extended our search to the preprint server arXiv; however, none of the non-peer-reviewed manuscripts retrieved met our inclusion criteria. The full protocol has been registered on Open Science Framework [8]. Risk of bias and applicability concerns were evaluated for each included study using the Quality Assessment of Diagnostic Accuracy Studies-2 (QUADAS-2) tool. Detailed ratings are provided in Supplementary Table S1. Full texts of eligible studies were assessed for relevance, with discrepancies resolved through consensus.

## 3. Results

### 3.1. Study Selection and Transparency

**Figure 1:**
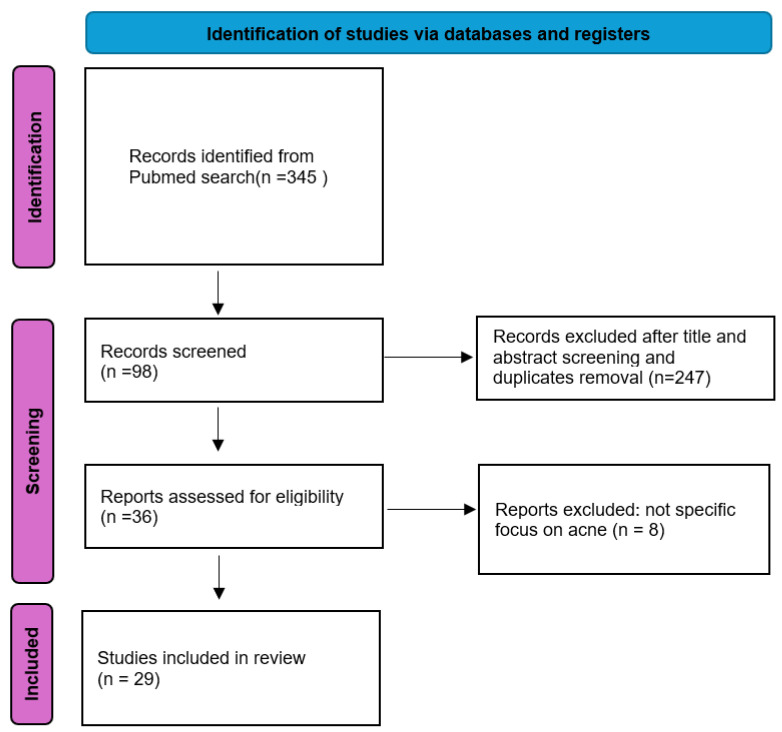
Our systematic review process following the Preferred Reporting Items for Systematic Reviews and Meta-Analyses (PRISMA) framework.

The systematic search identified 345 articles, with 98 studies undergoing full-text screening. Ultimately, 29 studies met inclusion criteria (Figure 1). The analysis focused on AI methodologies employed, datasets utilized, and the results achieved in acne diagnosis and severity grading. All the studies included in the review were retrospective. Among them, 13 (44.8%) relied solely on internal datasets, while 16 (55.2%) used publicly available datasets (ACNE04 [9] and AcneSCU [10]). Specifically, seven studies (24.1%) utilized only the ACNE04 dataset, one study (3.4%) used the AcneSCU dataset, five studies (20.7%) integrated ACNE04 with internal data, and one study (3.4%) combined ACNE04, AcneSCU, and internal data.

Additionally, one study utilized an AI-generated synthetic dataset, created by training a Generative Adversarial Network (GAN) on ACNE04 [11]. One study [11] (3.4%) used a public dataset of faces (CelebAMask-HQ and 2037 images from the Flickr-Faces-HQ), a minority of whom were acne-affected. It is worth noting that, while most of the studies have used images taken from outpatient clinics photos, seven studies (27.6%) used patient self-taken smartphone photos. Publicly accessible code was available in only four studies (13.8%).

### 3.2. Participant Demographics and Dataset Diversity

Participant age was reported in only five studies (17.2%). Information on participant ethnicity was available for 16 studies (55.2%), including those relying exclusively on the publicly available ACNE04 dataset. Among these, 15 studies (51.8%) primarily included individuals of East Asian or Chinese ethnicity. The only exception was the study by Seitè et al. [5] that analyzed 5972 images from 1072 acne patients representing different ethnic groups, including Caucasian, African, Asian, Latin American, and Indian populations, across countries such as France, South Africa, China, and India. Images were captured using both iOS and Android smartphones, ensuring broad representation of skin colors and types.

### 3.3. Algorithms

Among the 29 studies analyzed, 11 (37.9%) utilized multiple deep learning (DL) algorithms, resulting in a total of 47 distinct models. CNNs and deep convolutional neural networks (DCNNs) were the predominant architectures, accounting for 79% (23/29) of all models examined (Table 1). The primary research focus was acne lesion segmentation and counting (12 studies, 41.4%) and severity grading (16 studies, 55.2%), while only one study (3.4%) investigated binary classification (presence vs. absence of acne).

The training datasets exhibited substantial variability, ranging from 1213 to over 150,000 images. Key methodological approaches included CNN-based architectures, semi-supervised learning techniques, and generative adversarial networks (GANs), such as StyleGAN2-ADA, which were employed to generate synthetic datasets, thereby addressing data scarcity and enhancing model generalization. Training data were typically partitioned into training and validation subsets, with 80–90% allocated for training and the remainder reserved for validation.

Model validation was conducted using either k-fold cross-validation or holdout methods to ensure rigorous performance assessment. Metrics such as Dice coefficients, accuracy, and the area under the receiver operating characteristic (AUC-ROC) curve were commonly used to evaluate predictive performance, particularly in lesion detection and acne severity grading (Table 1). Despite the overall robust methodology, 17 studies (58.6%) did not provide detailed information on image resolution or validation set specifications, raising concerns about reproducibility. Reported accuracy varied widely, ranging from 20.5% (ACNet10) to 86.06% (DED framework via Knowledge Distillation [12]). Results are summarized in Table 2.

**Table 1:** Characteristics of the included studies.

Authors	Application Category	Methods	Dataset	Dataset Source	Publicly Available Code	Number of Photos (Total)
Shen et al. [13] (2017)	Automatic Acne Diagnosis	Binary classifier for skin detection and seven-class classifier for acne lesion classification, using CNNs with data augmentation techniques.	Binary classifier: 3000 cutaneous and 3000 non-cutaneous images. Seven-class classifier: 6000 images per class (blackheads, whiteheads, papules, pustules, cysts, nodules, normal skin).	Clinical images, augmented with rotation, shift, shear, scaling, and horizontal flip.	No	Not specified
Zhao et al. [14] (2019)	Acne Severity Assessment using Selfie Images	Deep learning model using transfer learning with ResNet 152 and a novel rolling image augmentation approach to improve CNN model generalization.	4700 selfie images, with 4470 used for training and 230 used for testing.	Selfie images collected by Nestlé SHIELD and labeled by 11 dermatologists.	Code available on GitHub: https://github.com/Microsoft/nestle-acne-assessment (accessed on 5 March 2025)	4700
Wu et al. [9] (2019)	Joint Acne Image Grading and Counting via Label Distribution Learning	Label Distribution Learning framework that integrates global grading and local lesion counting.	1457 facial images from the ACNE04 dataset with 18,983 annotated lesions.	Clinical images collected under the Hayashi grading criterion, taken at a 70-degree angle.	Code and dataset available on GitHub: https://github.com/xpwu95/ldl (accessed on 5 March 2025)	1457
Seitè et al. [5] (2019)	Acne Grading from Smartphone Photographs	AI Algorithm trained on a large and diverse dataset, using deep learning techniques for acne grading and lesion identification.	5972 images from 1072 patients with acne, including 2939 inflammatory lesions, 7603 non-inflammatory lesions, and 5702 PIHP instances.	Smartphone images (iOS and Android) across various racial groups.	No	5972
Lim et al. [15] (2019)	Automated Acne Grading using Deep Learning and CNNs	Deep learning models (DenseNet, Inception v4, ResNet18) trained on high-resolution images to automatically calculate IGA scores.	472 frontal facial images from 416 acne patients, with training set of 314 images and testing set of 98 images, augmented to 6248 images.	Clinical images captured at ~2000 × 3000 pixel resolution.	No	472
Peris Fajarnés et al. [16] (2020)	Segmentation Methods for Acne Vulgaris Images using Fluorescence Imaging	K-means clustering algorithm implemented in MATLAB R2018b for automated segmentation and counting of acne lesions in fluorescence images.	54 processed fluorescence images of mild acne patients, captured using iPhone X with Wood’s lamp and LED lamp.	Fluorescence images captured using iPhone X smartphone.	No	54
Rashataprucksa et al. [17] (2021)	Acne Detection	Faster R-CNN and R-FCN models used for acne detection, focusing on various types of acne lesions.	871 annotated images with 15,917 lesions categorized into four types: Type I, Type III, PIE, and PIH.	Clinical images provided by Pan Rajdhevee Group Public Co., Ltd.; Bangkok, Thailand.	No	871
Yang et al. [18] (2021)	Acne Vulgaris Assessment	Inception-v3 architecture used for model construction, trained on clinical images categorized into four severity grades.	5871 clinical images, with 1565 images in the training set, 392 in the validation set, and 40 in the test set.	Clinical images captured with single-lens reflex cameras.	No	5871
Quattrini et al. [12] (2022)	Facial Acne Classification	Used a VGG-19 based CNN for classification and BiSeNet for semantic segmentation, trained on re-annotated images from the FFHQ dataset.	2307 re-annotated images from the Flickr-Faces-HQ (FFHQ) dataset, used for acne severity classification.	High-resolution images from the FFHQ dataset, re-annotated for acne severity.	No	2307
Min et al. [19] (2022)	Acne Detection using Mask-Aware Attention with Dynamic Context Enhancement	ACNet integrates Composite Feature Refinement, Dynamic Context Enhancement, and Mask-Aware Multi-Attention for enhanced feature representation and detection accuracy.	ACNE04 dataset with 1457 facial images and 18,983 annotated lesions.	Clinical photographs with varying resolutions, annotated by dermatologists.	No	1457
Zhang et al. [20] (2022)	Acne Detection using High-Quality Proposals and Region Proposal Network	Spatial Aware Double Head for classification and localization, Normalized Wasserstein Distance (NWD) for accurate localization confidence prediction.	AcneSCU dataset with 276 high-resolution facial images and 31,777 annotations across 10 lesion categories.	Clinical photographs with high-resolution images, captured using the VISIA complexion analysis system.	Code and dataset available on GitHub: https://github.com/pingguokiller/acnedetection (accessed on 5 March 2025)	276
Zhang et al. [10] (2022)	Acne Detection	Ensemble neural network composed of ResNet50-based classification and YOLOv5-based localization modules for simultaneous severity, count, and bounding-box prediction	ACNE04: 1457 facial images with expert-annotated lesion counts, severity grades, and bounding boxes	ACNE04 dataset	No 1457	
Wang et al. [21] (2022)	Cell Phone App for Facial Acne Severity Assessment	Acne-RegNet, a lightweight model for lesion detection and classification, combined with metadata for personalized severity assessment.	1455 images from ACNE04 and 1515 images from a private dataset at Xiangya Hospital, processed into 80 × 80 lesion patches and resized for the model.	Clinical photographs, including metadata, collected from a private dataset and the ACNE04 dataset.	No	2970
Wang et al. [22] (2022)	Acne Detection and Severity Quantification	Two-stage deep learning scheme	1803 facial images (1374 for training/test, 429 for clinical validation)	Smartphone images captured with iPhone 5s at Xiangya Hospital, annotated by dermatologists	No	1803
Huynh et al. [23] (2022)	Automatic Acne Object Detection and Severity Grading Using Smartphone Images and AI	Faster R-CNN deep learning model for acne object detection and LightGBM machine learning model for grading acne severity based on the IGA scale.	1572 facial images with 41,859 labeled lesions, captured by iOS and Android smartphones.	Smartphone images collected via the ’Skin Detective’ mobile application.	No	1572
Lin et al. [24] (2022)	Unified Acne Grading Framework on Face Images	KIEGLFN framework with Key Information Enhancement and Global Local Fusion Network, including a transfer fine-tuning strategy for new grading criteria.	1457 images from the ACNE04 dataset, 200 images from the PLSBRACNE01 dataset, annotated for lesion location, severity, and type.	Clinical images from the ACNE04 and PLSBRACNE01 datasets.	Code available on GitHub: https://github.com/linyi0604/KIEGLFN (accessed on 5 March 2025)	1657
Liu et al. [25] (2022)	Ensemble Pruning of Deep Learning Base Models for Acne Grading	Ensemble pruning of deep learning models, fivefold cross-validation, and transfer learning applied to both acne and skin cancer datasets.	1457 images from the ACNE04 dataset, annotated for lesion location and severity grading; 3297 dermoscopic images in the skin cancer dataset, categorized as benign or malignant.	Clinical images from the ACNE04 and skin cancer datasets.	Datasets available at: https://github.com/xpwu95/ldl and https://www.kaggle.com/datasets/fanconic/skin-cancer-malignant-vs-benign (accessed on 5 March 2025)	4754
Wen et al. [26] (2022)	Acne Detection and Severity Evaluation	Interpretable CNN-based object detection models, with a focus on interpretability	1457 images from the ACNE04 dataset, with 1222 high-quality images used after data cleaning.	Clinical images with annotations for lesion location, severity, and bounding boxes.	Code available on GitHub: https://github.com/wenh06/acne_detection/ and https://github.com/wenh06/yolov4_acne_torch (accessed on 5 March 2025)	1457
Kim et al. [27] (2023)	Acne Lesion Detection and Counting for Severity Evaluation	CNN-based algorithm, with a focus on binary and five-class classification of acne lesions.	1213 images with 20,699 labeled lesions (closed comedones, open comedones, papules, nodules/cysts, pustules).	Clinical images acquired in standardized settings using Canon EOS 550D and NIKON D7100 cameras.	Detailed code and hyperparameters provided in the Electronic Supplementary Materials.	1213
Wei et al. [28] (2023)	Accurate Acne Detection via Decoupled Sequential Detection Head	Decoupled Sequential Detection Head mechanism applied to mainstream two-stage detectors.	ACNE-DET: 276 high-resolution images with 31,777 labeled lesions; ACNE04: 1457 images with 18,983 bounding box annotations.	Clinical images acquired with VISIA system and annotated by dermatologists.	Code and dataset are publicly available, repository not specified.	276
Li et al. [29] (2023)	AI-Powered Acne Grading System with Lesion Identification	ResNet50 architecture, with 945 images used for training and 240 for testing, incorporating lesion identification and severity grading.	1501 standardized photos from 1501 acne patients, with detailed labeling of 15,922 lesions across 10 categories.	Clinical images obtained using the VISIA complexion analysis system.	No	1501
Lin et al. [30] (2023)	Acne Severity Grading via Diagnostic Evidence Distillation	Teacher-student structure using CNNs for both global estimation and lesion detection, integrating lesion counting and grading.	ACNE04: 1457 facial images with 13,633 lesions; PLSBRACNE01: 200 images categorized by severity.	Clinical images labeled according to Hayashi and Pillsbury criteria.	Code available on GitHub: https://github.com/linyi0604/DED (accessed on 5 March 2025)	1457
Li et al. [6] (2023)	AI Analysis of Dark Circles and Facial Skin Aging in Chinese Population	CNNs based system for grading facial features from selfies, validated against dermatologist assessments.	Over 1.9 million selfies from 1,939,586 participants, graded for facial aging indicators.	Selfies taken with smartphones, collected via a mobile app.	No	1,939,586
Li et al. [31] (2024)	Acne severity assessment	CNN-based automatic grading	1,156,703 women aged 18–85	Selfies taken with smartphones, collected via a mobile app. Annotated by dermatologists.	No	1,156,703
Kim et al. [32] (2024)	Facial Acne Segmentation	Semi-supervised learning using bidirectional copy–paste and deep learning with U-Net.	Acne images cropped from facial images	Clinical images (likely taken with specialized skin diagnostic equipment)	No	2000
Zhang et al. [33] (2024)	Acne Detection using CenterNet	CenterNet-based deep learning framework, utilizing deep hierarchical aggregation strategies for feature extraction, combined with anchor-free detection for improved accuracy in locating and identifying acne lesions.	153,183 images collected from 15 hospitals in different provinces of China using smartphones.	A mix of clinical and smartphone images	No	153,183
Prokhorov et al. [34] (2024)	Acne Image Grading using Label Distribution Smoothing	Gaussian label distribution generation and label smoothing techniques combined with a scale-adaptive approach, validated on the ACNE04 dataset using ResNet-50 architecture.	ACNE04 dataset with 1457 images and 18,983 bounding boxes of lesions.	Clinical images from the ACNE04 dataset, specifically annotated for acne grading.	Source code is publicly available at http://github.com/openface-io/acne-lds	1457
Zein et al. [11] (2024)	Acne Dataset Generation Using Generative Adversarial Networks (GANs)	GAN-based method with StyleGAN2 to generate synthetic acne images at different severity levels, used for training a CNN-based classification system.	1473 real images initially collected, with additional synthetic images generated across Mild, Moderate, and Severe severity levels.	Synthetic images generated using GANs based on clinical images.	Code and dataset available on GitHub: https://github.com/hz01/AcneGAN/tree/main	1473
Gao et al. [35] (2025)	Acne lesion detection and severity grading in online and offline scenarios	Deep learning model (AcneDGNet) that combines a vision transformer–based feature extractor with a CNN-based lesion detection module and a severity grading module	2157 facial acne images compiled from two public datasets (ACNE04, AcneSCU) and three self-built datasets (AcnePA1, AcnePA2, AcnePKUIH)	Mixed capture sources: digital cameras, VISIA system, and smartphone images	No	2157

**Table 2:** Results of the included studies.

Study	Task	Models	Dataset	Validation	Metrics
Shen et al. (2018) [13]	Binary acne detection and 7-class acne classification	Custom CNN; pre-trained VGG16	Binary: 3000 skin + 3000 non-skin patches; Seven-class: 6000 patches per class (7 classes); Not specified	Train/validation/test split (80/10/10%) for both classifiers	Binary acne detection: accuracy 92%, 7-class acne classification: per-class accuracy > 81%
Lim et al. (2019) [15]	Investigator’s Global Assessment (IGA) grading	CNN architectures (DenseNet, Inception v4, ResNet18)	472 frontal images (374 train, 60 val, 98 test), augmented to 6248 train, 1200 val; Not specified	Hold-out validation	Best classification accuracy 67%
Seité et al. (2019) [5]	Global Acne Severity (GEA) grading and lesion segmentation	Not specified	5972 smartphone photos	Not specified	GEA grading accuracy 68%;
Zhao et al. (2019) [14]	Acne severity assessment from selfies	ResNet-152 pre-trained + fully connected regression CNN	4470 training images; 230 test images;	Hold-out golden set labeled by 11 dermatologists	Root-mean-square error 0.482 (baseline 0.78; without augmentation 0.72);
Wu et al. (2019) [9]	Joint acne grading and lesion counting	Label Distribution Learning; CNN backbone (ResNet-50)	1457 images (1165 train; 292 test); ACNE04 dataset	5-fold cross-validation	Accuracy 84.11%;
Rashataprucksa et al. (2020) [17]	Acne lesion detection	Faster R-CNN; R-FCN	871 annotated facial images (783 train; 88 test);	Hold-out test set evaluation	Mean average precision: Faster R-CNN 0.233; R-FCN 0.283
Peris Fajarnés et al. (2020) [16]	Acne lesion segmentation and counting	k-means clustering segmentation	Fluorescence images of mild acne patients; validation on 54 images; Fluorescence imaging (Wood’s lamp and LED with filters)	Comparison to manual expert counts	Segmentation effectiveness > 90%; discrepancy ≤ 11.2%
Yang et al. (2021) [18]	Acne severity grading	Inception-v3 CNN (transfer learning)	5871 clinical images (1565 train; 392 val; 40 test); Clinical photos, Chinese Academy of Medical Sciences	Hold-out test set against attending dermatologists	Average F1 = 0.80; weighted kappa = 0.791
Zhang et al. (2022) [20]	High-quality proposal generation for acne detection	Spatial Aware Double Head and NWD prediction on RPN	276 images; 31,777 annotations; AcneSCU dataset	90/10 train:test split; separate patients	Improved proposal AR and detection mean average precision (specifics N/A)
Zhang et al. (2022) [10]	Acne severity classification and lesion localization	Ensemble NN with ResNet50 classifier and YOLOv5 localization	1457 facial images; ACNE04 public dataset	Hold-out test set (80/20 split)	Best accuracy 90.67%
Lin et al. (2022) [24]	Unified acne grading across criteria	KIEGLFN (Key Information Enhancement + GLFNet)	1457 ACNE04 images + 200 other images (PLSBRACNE01 dataset)	5-fold CV on ACNE04; 80/20 split on PLSBRACNE01	Accuracy 84.52% on ACNE04; 59.35% on PLSBRACNE01
Wang et al. (2022) [22]	Smartphone-based acne severity assessment	Acne-RegNet (lightweight CNN with attention)	1455 ACNE04 images + 1515 internal Xiangya Hospital images	Hold-out evaluation	Severity accuracy 94.56% on internal dataset
Liu et al. (2022) [25]	Acne grading via ensemble pruning	Ensemble pruning of deep learning base models (CNNs)	1457 ACNE04 images; 3297 skin cancer dermoscopic images; ACNE04 dataset; public skin cancer dataset (dermoscopic)	5-fold cross-validation on ACNE04; hold-out test on cancer dataset	Accuracy 85.82% on ACNE04
Wen et al. (2022) [26]	Acne lesion detection and severity evaluation	Faster R-CNN (ResNet101) and other interpretable CNNs	1222 images (Filtered ACNE04 dataset)	Not reported	mAP = 0.536; MAE = 3.49
Huynh et al. (2022) [23]	Acne lesion detection and severity grading	Faster R-CNN (ResNet50 backbone) for detection; LightGBM for grading	1572 smartphone facial images	Hold-out evaluation	mAP = 0.54 (detection); accuracy = 0.85 (grading)
Min et al. (2022) [19]	Acne lesion detection	ACNet (Composite Feature Refinement + DCE + MAMA)	ACNE04 public dataset	80/20 hold-out split	Detection mAP = 20.5%
Quattrini et al. (2022) [12]	Acne classification and semantic segmentation	Semantic Segmentation + DenseNet121	2307 FFHQ images re-annotated; 30,000 CelebAMask-HQ masks	Hold-out test set for segmentation and classification	Segmentation F1 avg 86.1%; Classification F1 avg 60.84%
Wang et al. (2023) [21]	Acne detection and severity quantification	Localization-DL (SE-ResNet-50 teacher + MobileNetV2 student) and ClassSeg pipelines	1374 self-collected clinical facial images	Hold-out test set; ablation and comparison vs. dermatologists	Severity quantification accuracy 90.91% vs. dermatologists (93.01%, 87.41%, 74.83%)
Kim et al. (2023) [27]	Automated lesion detection and counting for severity evaluation	PP-YOLO (improved YOLOv3) for five-class and binary lesion detection	1213 images; 20,699 manually labeled lesions; Clinical facial acne photography sets	Hold-out test set evaluation and reader test	Binary detection mAP 28.48%
Li et al. (2023) [29]	AI-powered acne severity grading incorporating lesion identification	ResNet-50 with fixed and learnable lesion-count integration	1501 VISIA images (276 for lesion ID, 1185 for grading); Standardized VISIA clinical photos from 1501 acne patients	Hold-out test sets	Lesion identification: precision 0.507, Recall 0.775; severity kappa: baseline 0.652, fixed weights 0.737, learnable weights 0.696
Wei et al. (2023) [28]	Fine-grained acne lesion detection	Decoupled Sequential Detection Head (DSDH) on two-stage detectors	Internal dataset of 276 images (241 train/35 test) and 31,777 lesion instances; + ACNE04	Hold-out test set on ACNE-DET	ACNE-DET—AP 45.6%, APS 44.3%, APM 46.9%, APL 34.1% (best config)
Lin et al. (2023) [30]	Acne severity grading across varied criteria	Diagnostic Evidence Distillation (DED) teacher–student CNN framework	ACNE04: 1457 images + 200 images	Hold-out test sets on both datasets	ACNE04—Precision 85.31%, Accuracy 86.06%;
					PLSBRACNE01—Precision 69.16%, Accuracy 67.56%
Li et al. (2023) [6]	Automatic grading of facial signs including acne vulgaris	ResNet50V2 transfer learning	1,939,586 selfie images (100,589 men; 1,838,997 women) from a smartphone app	Internal validation against dermatologist scores	Dark circles classification accuracy 91.5%; acne vulgaris grading metrics not separately reported
Zhang et al. (2024) [33]	Multi-task acne detection (quality control, classification, localization, segmentation)	CenterNet with DLA34 backbone and custom segmentation submodule	153,183 acne lesion images (150,219 train; 2322 val); 642 independent clinical test images; smartphone and clinical images from 15 hospitals in China (Jan 2020–Oct 2022)	Independent clinical test set; comparison against ResNet18 and dermatologists	Lesion categorization accuracy 83%; lesion stratification precision 76%
Li et al. (2024) [34]	Epidemiological analysis of adult female acne severity	ResNet-50 + FPN + RPN + ROI head CNN	1,156,703 high-res smartphone selfies from a smartphone app	8:1:1 train:val:test split; physician consensus on 100 samples	Inter-rater ICC 0.887; intra-rater ICC 0.900
Kim et al. (2024) [32]	Facial acne lesion segmentation	Semi-supervised U-Net with bidirectional copy–paste framework	Proprietary facial acne image dataset	Comparison with previous SSL methods across varying labeled proportions	Dice score 0.5205 with 3% labeled images (improvement 0.0151–0.0473)
Zein et al. (2024) [11]	Synthetic acne face dataset generation and CNN classification	StyleGAN-based GANs; InceptionResNetV2 classifier	ACNE04 public dataset	Not specified	Accuracy 97.6%
Prokhorov et al. (2024) [34]	Acne image grading with scale-adaptive label distribution smoothing	Scale-adaptive LDL + weighted label smoothing	ACNE04 public dataset	5-fold cross-validation	Accuracy 84.11 ± 1.94%; Precision 83.11 ± 2.56%;
Gao et al. (2025) [22]	Acne lesion detection and severity grading in online and offline scenarios	AcneDGNet (Swin Transformer + CNN modules)	ACNE04 + AcneSCU + internal datasets (AcnePA1, AcnePA2, AcnePKUIH)	Hold-out evaluations: online (AcnePA1 and PA2) and offline (ACNE04, AcneSCU, AcnePKUIH);	Online accuracy 89.5%; offline accuracy 89.8%;

## 4. Discussion

AI-based systems for acne grading are expected to improve dermatological care by providing standardized assessments, reducing inter-observer variability, and enhancing clinical efficiency. However, challenges such as data imbalance, limited dataset diversity, and difficulties integrating these tools into existing clinical workflows continue to limit their wider adoption.

The widespread application of AI-based acne grading might standardize dermatological research, making studies conducted in different regions and clinical settings more comparable and facilitating the development of therapeutic strategies based on robust and reproducible data. Furthermore, AI’s ability to process vast amounts of data from diverse skin types can enhance our understanding of acne’s incidence and geographical distribution. It can also help identify new risk factors, support the development of updated epidemiological registries, and contribute more effectively to health policy. Li et al. [6] used AI to analyze facial acne in adult women across China, involving 1,156,703 participants. The study employed a smartphone application, “You Look Good Today,” to collect high-resolution selfies and user data through questionnaires, including information on age, skin sensitivity, dietary habits, and environmental factors. The AI algorithm, based on a ResNet-50 architecture combined with a Feature Pyramid Network and a Region Proposal Network, classified acne severity into four grades. The Overall Severity Score (OSS) was calculated using the following formula: *OSS* = 100 × (∑(*Si*^2^))^1/2^.

In this formula, *Si* represents the severity score assigned to each acne lesion detected by the CNN in a selfie. The scores were defined as follows: 1 for comedones only, 2 for papules, 3 for pustules, and 4 for nodules and cysts. The study revealed that the OSS decreases from age 25, reaches its lowest point between ages 40 and 44, and then gradually increases again. The analysis indicated that oily and sensitive skin, frequent use of cosmetic products, and unhealthy dietary habits significantly contribute to higher acne severity. Environmental factors, such as the level of urban development, season, altitude, and solar radiation, also impacted acne severity, with more severe acne observed in developed cities and during autumn and winter seasons [6]. The study further found a positive correlation between acne severity and other facial issues, such as enlarged pores, blackheads, skin roughness, and dark circles [6].

However, while some models have demonstrated high accuracy in controlled environments [35], translating these results into clinical practice remains challenging: to date, none of the proposed algorithms has received clinical validation and no study has been published regarding real-life settings. Prospective studies with real-world testing are needed to validate the effectiveness of these AI systems in routine dermatological care.

Unlike other dermatological conditions, which have extensive public databases supporting standardized AI algorithm development, acne remains underrepresented in these resources [36]. Privacy concerns surrounding the public sharing of images of acne-affected faces continue to be a major obstacle to building large, publicly accessible datasets. Consequently, most datasets used in acne-related studies are not publicly accessible [12].

Quattrini et al. [12] used a public dataset of faces: Flickr-Faces-HQ (FFHQ), consisting of 70,000 high-quality PNG images of human faces at 1024×1024 resolution and containing considerable variation in terms of age, ethnicity and image background. However, because most images did not show acne, the dataset was highly unbalanced: about 88% of the images fell into the “No Acne” category. This imbalance restricted the study to simply detecting the presence of acne rather than assessing its severity.

The ACNE04 dataset is a notable exception [30]. This public dataset contains 1457 facial images with acne annotations, each labeled with acne severity and lesion count by professional dermatologists. However, the dataset has several limitations, including an imbalance in lesion counts, with most images containing fewer than 10 lesions and few images with high lesion counts (40 to 50), and it consists solely of Han Chinese subjects [30].

Similarly, another public resource is represented by the AcneSCU dataset [10], which includes 276 annotated facial images of acne, but its limited size and focus solely on Han Chinese patients also constrain its broader applicability.

A novel approach to address the challenges posed by the limited availability of public acne datasets has been recently proposed [11] using GANs to create AI-generated synthetic and anonymized datasets of acne-affected faces. In this study, a small initial dataset consisting of 1473 images from the ACNE04 dataset and additional sources was pre-processed and augmented to generate high-resolution synthetic images (1024 × 1024) representing three levels of acne severity: mild, moderate, and severe. By generating realistic images with different grades of acne severity, the study not only avoids the ethical and legal challenges associated with sharing medical images but also contributes to the diversification of available data, as the synthetic datasets are not confined to any specific ethnicity or lesion distribution. The generated datasets were then used to train three different CNNs (InceptionResNetV2, ResNet50V2, and ResNet152V2) solely on synthetic images. Then, real acne images were used to test these models. The best-performing model, InceptionResNetV2, achieved a grading accuracy of 97.6% when validated on real images, demonstrating the viability of this approach for future research on this topic. It should also be noted that the accuracy of the estimation of acne severity may be influenced by lighting conditions, facial expressions, and individual variations in skin type [13].

Ethnic diversity also remains another major limitation: the two largest public image datasets (ACNE04 and AcneSCU) comprise only Han Chinese subjects, and most published studies focus on East Asian individuals. In other dermatology applications, lack of skin-type diversity has been shown to reduce algorithmic accuracy on under-represented group in training sets [3]: acne detection and grading models trained predominantly on East Asian data are likely to underperform in other ethnic populations.

Another aspect revealed by this review, and a further obstacle for the real-life applications of automatized acne grading systems, is the pervasive lack of transparency—58.6% of studies failed to report crucial details such as image resolution or the makeup of their independent validation sets, severely undermining both reproducibility and inter-study comparability. Moreover, only 13.8% have made their source code publicly available, and nearly half (44.8%) rely exclusively on internal, non-public datasets. Practical implementation of external validation could involve a hold-out set drawn from partner dermatology clinics, with subsequent cross-site performance benchmarking and domain-adaptation techniques to identify and mitigate dataset biases. Addressing these shortcomings through open data, shared code, and prospective, multicenter clinical trials will be crucial to developing unbiased, generalizable tools.

Other crucial issues include data privacy and security (transmitting sensitive facial images), data interoperability (integrating AI outputs into electronic health records or telehealth systems) and regulatory approval. In future, privacy-preserving methods like federated learning may help address confidentiality concerns [37]. Ultimately, adoption will depend on evidence of benefit in real-world workflows and compliance with evolving regulations and ethical guidelines.

In conclusion, to date none of the published algorithms have demonstrated the generalizability, reproducibility, or external and prospective clinical validation necessary for integration into routine dermatological practice. Future work in AI-driven acne diagnosis and severity grading should address these gaps, particularly the need for real-world prospective clinical trials to evaluate model performance in real-world settings [35]; the creation of large, publicly accessible, and demographically diverse imaging repositories [11]; the exploration of federated learning to enable privacy-preserving cross-institutional model training; and the integration of explainable AI methods to enhance clinician interpretability and adoption.

Despite current limitations, however, the results are encouraging: AI-driven tools promise to transform acne research and clinical care, reducing inter- and intra-observer variability and enabling truly personalized, timely therapies. By refining model architectures, broadening and diversifying available datasets, and rigorously validating performance in real-world settings, the field can unleash AI’s full potential in this condition.

## Data Availability

The original contributions presented in this study are included in the article/Supplementary Materials. Further inquiries can be directed to the corresponding author.

## Supplementary Materials

The following supporting information can be downloaded at https://www.mdpi.com/article/10.3390/jpm15060238/s1, Table S1: QUADAS2 assessment of the included studies.

## Abbreviations

The following abbreviations are used in this manuscript:

IGA	Investigator Global Assessment
AI	Artificial Intelligence
CNN	Convolutional Neural Network
GAN	Generative Adversarial Networks
OSS	Overall Severity Score
