# Supplementary material for: Artificial Intelligence in the Assessment and Grading of Acne Vulgaris: A Systematic Review
Source: J Pers Med. 2025 Jun 6;15(6):238. doi: 10.3390/jpm15060238 (PMC12194645; doi:10.3390/jpm15060238)
Supplement: Supplementary file 1 [file jpm-15-00238-s001.zip › jpm-3618123-supplementary.pdf]

Supplementary Table S1. QUADAS2 assessment of the included studies

Study	Risk of Bias: Patient Selection	Risk of Bias: Index Test	Risk of Bias: Reference Standard	Risk of Bias: Flow and Timing	Applicability: Patient Selection	Applicability: Index Test	Applicability: Reference Standard
Shen et al. (2018) [13]	Undefined	Undefined	Undefined	Undefined	Yes	Yes	Yes
Zhao et al. (2019) [14]	Undefined	Undefined	Undefined	Yes	Yes	Yes	Yes
Wu et al. (2019) [9]	Undefined	Yes	Yes	Yes	Yes	Yes	Yes
Seité et al. (2019) [5]	Undefined	Undefined	Undefined	Undefined	Yes	Yes	Yes
Lim et al. (2019) [15]	Undefined	Undefined	Yes	Yes	Yes	Yes	Yes
Peris Fajarnés et al. (2020) [16]	Undefined	Undefined	Undefined	Yes	Yes	Yes	Yes
Rashatapprucksa et al. (2020) [17]	Undefined	Undefined	Undefined	Undefined	Yes	Yes	Yes
Yang et al. (2021) [18]	Undefined	Yes	Yes	Undefined	Yes	Yes	Yes
Quattrini et al. (2022) [12]	Undefined	Undefined	Undefined	Undefined	Yes	Yes	Yes
Min et al. (2022) [19]	Undefined	Yes	Yes	Yes	Yes	Yes	Yes
Zhang et al. (2022) [20]	Undefined	Undefined	Undefined	Undefined	Yes	Yes	Yes
Zhang et al. (2022) [10]	Undefined	Undefined	Undefined	Undefined	Yes	Yes	Yes
Wang et al. (2022) [21]	Undefined	Undefined	Undefined	Yes	Yes	Yes	Yes
Huynh et al. (2022) [22]	Undefined	Undefined	Yes	Yes	Yes	Yes	Yes
Lin et al. (2022) [24]	Undefined	Undefined	Undefined	Yes	Yes	Yes	Yes
Liu et al. (2022) [25]	Undefined	Undefined	Undefined	Yes	Yes	Yes	Yes
Wen et al. (2022) [26]	Undefined	Undefined	Undefined	Undefined	Yes	Yes	Yes
Kim et al. (2023) [27]	Undefined	Undefined	Yes	Undefined	Yes	Yes	Yes
Wei et al. (2023) [28]	Undefined	Undefined	Undefined	Yes	Yes	Yes	Yes
Li et al. (2024) [34]	Undefined	Undefined	Undefined	Undefined	Yes	Yes	Yes
Li et al. (2023) [30]	Undefined	Yes	Yes	Yes	Yes	Yes	Yes
Wang et al. (2023) [21]	Undefined	Undefined	Undefined	Undefined	Yes	Yes	Yes
Lin et al. (2023) [30]	Undefined	Undefined	Undefined	Yes	Yes	Yes	Yes
Li et al. (2024) [34]	Undefined	Undefined	Undefined	Undefined	Yes	Yes	Yes
Kim et al. (2024) [32]	Undefined	Undefined	Undefined	Yes	Yes	Yes	Yes
Zhang et al. (2024) [33]	Undefined	Undefined	Undefined	Undefined	Yes	Yes	Yes
Zein et al. (2024) [11]	Undefined	Undefined	Undefined	Undefined	Yes	Yes	Yes
Prokhorov & Kalinin (2024) [34]	Undefined	Yes	Undefined	Yes	Yes	Yes	Yes
Gao et al. (2025) [22]	Undefined	Undefined	Undefined	Yes	Yes	Yes	Yes
